# Stratification of the immunotypes of tongue squamous cell carcinoma to improve prognosis and the response to immune checkpoint inhibitors

**DOI:** 10.1007/s00262-025-03982-9

**Published:** 2025-03-01

**Authors:** Yuya Su, Ryo Ouchi, Pissacha Daroonpan, Miwako Hamagaki, Tohru Ikeda, Noji Rika, Naoto Nishii, Fumihiko Tsushima, Yoshihito Kano, Takahiro Asakage, Makoto Noguchi, Hiroyuki Harada, Miyuki Azuma

**Affiliations:** 1 Department of Oral and Maxillofacial Surgical Oncology, Graduate School of Medical and Dental Sciences, Institute of Science Tokyo (SCIENCE TOKYO), 1-5-45 Yushima, Bunkyo-Ku, Tokyo, 113-8519 Japan; 2 Oral Science Center (OSC), Institute of Science Tokyo (SCIENCE TOKYO), Tokyo, 113-8519 Japan; 3 Department of Molecular Immunology, Graduate School of Medical and Dental Sciences, Tokyo Medical and Dental University (TMDU), Tokyo, 113-8549 Japan; 4 Department of Oral and Maxillofacial Surgery, Graduate School of Medicine and Pharmaceutical Sciences for Research, University of Toyama, Toyama, 930-0194 Japan; 5 Department of Oral Diagnosis, Naresuan University, Phitsanulok, 65000 Thailand; 6 Division of Surgical Pathology, Institute of Science Tokyo Hospital, Tokyo, 113-8519 Japan; 7 Department of Oral Pathology, Graduate School of Medical and Dental Sciences, Institute of Science Tokyo (SCIENCE TOKYO), Tokyo, 113-8519 Japan; 8 Department of Medical Oncology, Graduate School of Medical and Dental Sciences, Institute of Science Tokyo (SCIENCE TOKYO), Tokyo, 113-8519 Japan; 9 Department of Head and Neck Surgery, Graduate School of Medical and Dental Sciences, Institute of Science Tokyo (SCIENCE TOKYO), Tokyo, 113-8519 Japan

**Keywords:** Oral cancer, Immune checkpoint inhibitor, Immunotherapy, Immunotypes, Tumor microenvironment

## Abstract

**Objectives:**

An understanding of the tumor immune microenvironment is required to improve treatment, especially the selection of immune checkpoint inhibitors (ICIs). In this study, we stratified the immunotypes of tongue squamous cell carcinoma (TSCC) based on the results of comprehensive immune profiling.

**Methods:**

We enrolled 87 therapy-naïve TSCC and 17 ICI-treated TSCC patients who underwent glossectomy without any other prior therapy. Comprehensive immune profile analyses employed multiplex immunofluorescence and tissue imaging.

**Results:**

Based on the hierarchies of 58 immune parameters and the spatial distances between cytotoxic T lymphocytes (CTL) and tumor cells, we stratified five immunotypes: Immunoactive type I, border type II, immunosuppressed type III, immunoisolating type IV, and immunodesert type V. The type I frequency was only 16%. Most TSCCs (~ 70%) were of types III–V. The CTL density (CTL-D) was closely correlated with the PD-L1^+^ pan-macrophages (panM)-D, and the panM-D closely correlated with the PD-1^+^ CTL-D. This indicated that PD-1 and PD-L1 expression required macrophages and CTL recruitment in the tumor microenvironment. No ICI-treated TSCC patients, all of whom were recurrent/metastatic cases, were of the type I immunotype, and almost half (47.0%) were of the immunodesert type V. Most cases exhibited an imbalance between T-cell PD-1 and macrophage PD-L1 expression.

**Conclusion:**

We defined five TSCC-specific immunotypes based on the results of comprehensive immune profiling analyses. Immunoactive type, which would be sensitive to ICI monotherapy, was rare, and most TSCC cases exhibited immune-regulated immunotypes. Immunotype-based personalized treatments are required to improve clinical outcomes.

**Supplementary Information:**

The online version contains supplementary material available at 10.1007/s00262-025-03982-9.

## Introduction

Anti-cancer immune responses play roles in the generation, progression, and eradication of cancer [1]. Many types of immune cells, including T and B cells, natural killer cells, macrophages, and dendritic cells, both positively and negatively contribute to the above processes. Although the current guidelines for cancer treatment are based on the tumor–node–metastasis (TNM) staging system and the pathological results, appropriate evaluation of the immune contexture or the immunoscore is required to improve the prognosis and therapeutic responses, especially at the choice of immunotherapies such as immune checkpoint inhibitors (ICIs) [2].

ICIs targeting programmed cell death 1 (PD-1) have been approved for the treatment of recurrent and metastatic (R/M) head and neck squamous cell carcinoma (HNSCC) in both first- and second-line settings and a consensus statement on immunotherapy (ICIs) for the treatment of R/M HNSCC has been proposed by the Society for Immunotherapy of Cancer (SITC) [3]. Unfortunately, unlike non-small cell lung cancers including lung SCC, which exhibit a good PD-1 ICI response [4], only 10% of PD-1 ICI-treated HNSCC demonstrated a durable long-term response [5].

Presently, several proposals of immunotype classification in HNSCC have been reported [6, 7]. Three tongue SCC (TSCC) immunogene signatures were identified: cold (no immune infiltrate), lymphocytes (CD4^+^ and CD8^+^ T cells, B cells, and plasma cells), and myeloid/dendritic cells (myeloid cells, regulatory T cells [Tregs], and eosinophils). These partly correlated with tumor–node factors and survival [6]. Analyses of 29 human papillomavirus (HPV)-negative HNSCC samples revealed four immunotypes based on CD8^+^ T cell density: fully infiltrated, stroma-restricted, immune-excluded, and immune-desert [7], all of which were independent of the tumor characteristics, T- and disease-stages, recurrence, and extra nodal extension (ENE) status. HNSCC involves diverse anatomical sites including the oral cavity, pharynx, and larynx. Anatomical differences influence the local anti-tumor immune responses. Recent comparative analyses of oropharyngeal, oral cavity, hypopharyngeal, and laryngeal SCCs using flow cytometry and single-cell RNA sequencing revealed that oral cavity SCC exhibited the highest levels of T cells [8]. However, these methods did not yield the densities of infiltrating cells or the spatial cell distributions. Changes in the magnitude of immune cell infiltration in the tumor microenvironment (TME) render frequency evaluation inappropriate. Oral cavity SCC occur in the tongue, gingiva, buccal mucosa, hard palate, floor of tongue, and lips. The site-specific parameters that influence oncogenesis including HPV status and tobacco/alcohol consumption affect the tumor immune microenvironment (TIME) [9, 10].

TSCC is the most common in the oral cavity and possess unique TIME [11]. We recently focused on TSCC and performed comprehensive immune profiling using multiplex immunofluorescence (mIF) and tissue imaging [12]. We demonstrated that the individual TSCC immune profiles were highly diverse and could not be predicted using only the TNM and/or pathological classifications. In addition, we also found that the cases exhibiting abundant immune cell infiltration were very rare. Most patients had low densities of leukocytes and CD8^+^ T cells. Therefore, TSCC-specific immunotype classification, that focus on the immune regulated mechanisms, is required for appropriate treatment selection including ICI and additional combined therapies.

After the development of PD-1 ICIs to treat R/M HNSCC, the clinicopathological and prognostic significance of PD-1 and PD-L1 expression were studied, but most studies used a cut-off value or a percentage for evaluation [13, 14]. No comprehensive assessment including immune cell subsets has been performed. The tumor proportion score (TPS) and combined positive score (CPS) for PD-L1 were not reliable biomarkers of an ICI response in patients with head and neck cancer [15, 16]. By gene-expression profile analyses, high immune cell infiltration scores serve as an effective prognostic biomarker and predictive indicator for immunotherapy in HNSCC [10].

In this study, we stratified TSCC immunotypes based on comprehensive immune profiling of total 87 therapy-naïve TSCC patients and defined correlations between immune cell subsets and PD-1/PD-L1 expression by immunotype. We then investigate the immune profiles and immunotypes of 17 PD-1 ICI-treated TSCC patients.

## Materials and methods

### Patients

The study design and methods were approved by the Institutional Review Board of the Institute of Science Tokyo (Tokyo Medical and Dental University) (#D2019-036). All methods and experiments were conducted in accordance with relevant guidelines and regulations. Informed consent was obtained with opt-out notice posted on the hospital bulletin board and website. In addition to the 60 previously reported therapy-naïve TSCC cases [12], we enrolled additional 27 TSCC patients who underwent glossectomy without prior therapy from 2010 to 2012 at Tokyo Medical and Dental University Hospital. The PD-1 ICI-treated cases were 21 TSCC patients who received nivolumab or pembrolizumab monotherapy, or pembrolizumab plus chemotherapy (5-fluorouracil plus platinum), at Tokyo Medical and Dental University Hospital from 2018 to 2021. Four patients who received chemotherapy and/or radiotherapy before glossectomy were excluded. Finally, the immune profiles of the initial surgical specimens of 17 patients were analyzed. We obtained clinicopathological information as described previously [12]. ICI therapy outcomes were evaluated based on the best overall response. Progression-free survival (PFS) was defined as the time from the initiation of ICI to the occurrence of disease progression. Tumor response was evaluated using Response Evaluation Criteria in Solid Tumors (RECIST) version 1.1 [17].

### Multiplex immunofluorescence and tissue image analysis

Selection of FFPE blocks and tissue sectioning were described previously [12]. Multiplex immunofluorescence (mIF) and tissue image analysis were performed using Opal mIF kits (Akoya Biosciences, Marlborough, MA) and a quantitative pathology imaging system (Mantra 1.0.3; PerkinElmer, Waltham, MA, USA) running Mantra Snap v1.0 and inForm ver. 2.4.11 software, respectively, as described previously [12]. Immune phenotyping proceeded as follows: CD45^+^CD8^+^ (cytotoxic T lymphocytes, CTL); CD45^+^CD4^+^Foxp3^–^ (conventional CD4^+^ T cells, Tcon); CD45^+^CD4^+^Foxp3^+^ (regulatory T cells, Treg); PD-1^+^ CTL, PD-1^+^ Tcon, and PD-1^+^ Treg, CD45^–^CK^+^ (tumor cells, Tu); PD-L1^+^ Tu; CD45^+^CK^–^ (leukocytes, Leu); CD45^+^CD68^+^ (pan-macrophages, panM); CD45^+^CD68^+^CD163^–^ (M1-like macrophages, M1); CD45^+^CD68^+^CD163^+^ (M2-like macrophages, M2); PD-L1^+^ M1, PD-L1^+^ M2, and CD45^+^CD66b^+^ (neutrophils, Neu); and non-T and non-myeloid CD45^+^ cells (Others). The density of each cell phenotype was determined as described previously [12].

For spatial assessment of CTL–tumor distance, the phenoptr package (Akoya Biosciences) running inForm software was used. The mean of top 100 shortest distances between CTL (CD45^+^CD8^+^DAPI^+^) and the nearest tumor cells (CK^+^DAPI^+^) (100 CTL-nT) was assessed for each image.

### Statistical analyses

We performed a comprehensive statistical assessment of all immune parameters using GraphPad Prism (ver. 10.1.1, GraphPad, San Diego, CA) and R (ver. 4.4.1) with RStudio software. For multiple comparison, the non-parametric Kruskal–Wallis test was conducted, followed by the uncorrected Dunn test for two group comparison in the case that a *p*-value of Kruskal–Wallis test (*p*_*k*_*)* was less than 0.05. For two group comparison, the Mann–Whitney U test was conducted. *p* < 0.05 was considered to indicate statistical significance. Correlation analyses were performed using Spearman’s rank correlation.

## Results

### Correlations among the hierarchies of 58 immune parameters

**Fig. 1:**
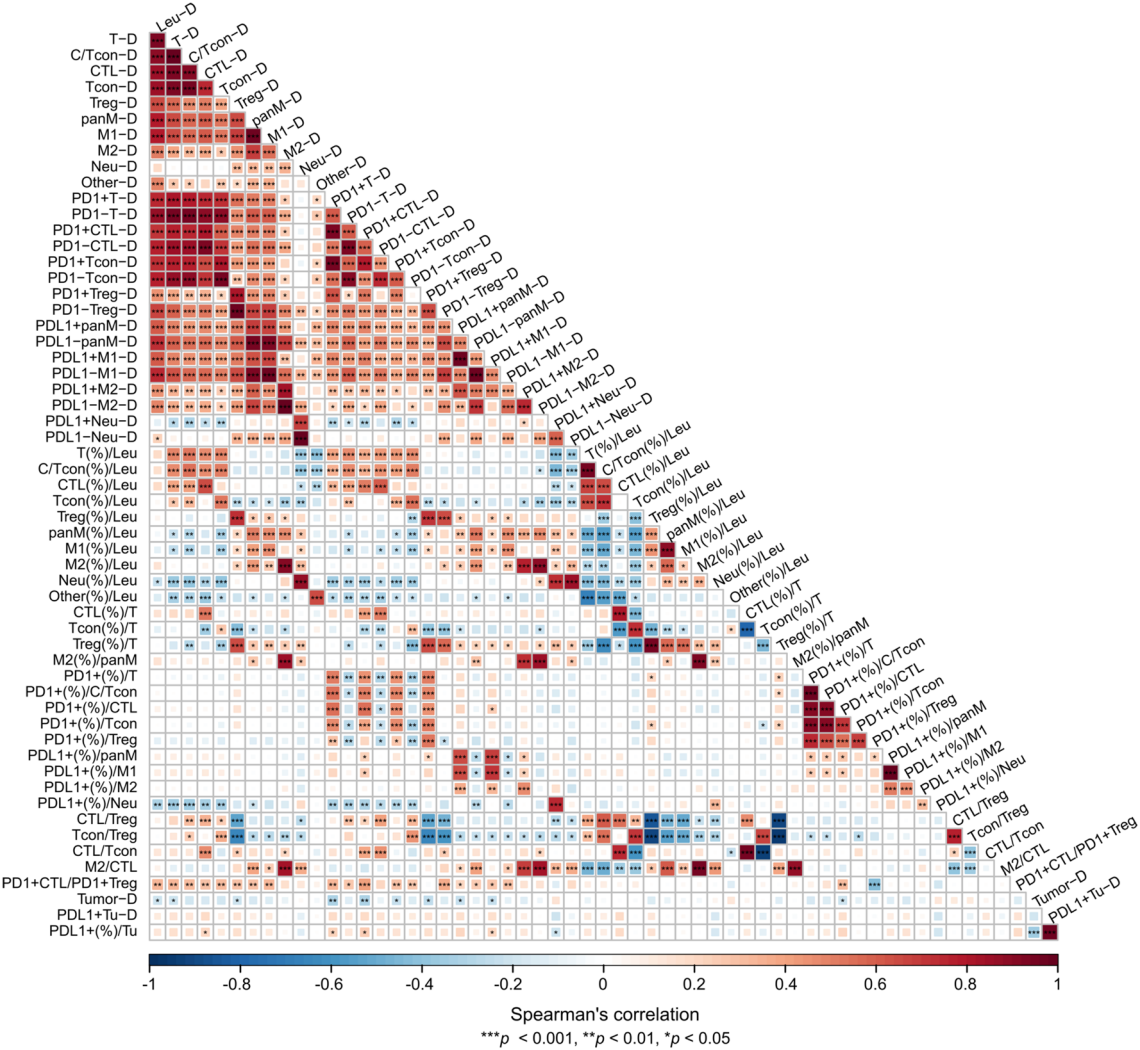
Correlations among 58 immune parameters. Spearman’s rank correlations are shown. The graded color band indicates the correlation coefficient

**Fig. 2:**
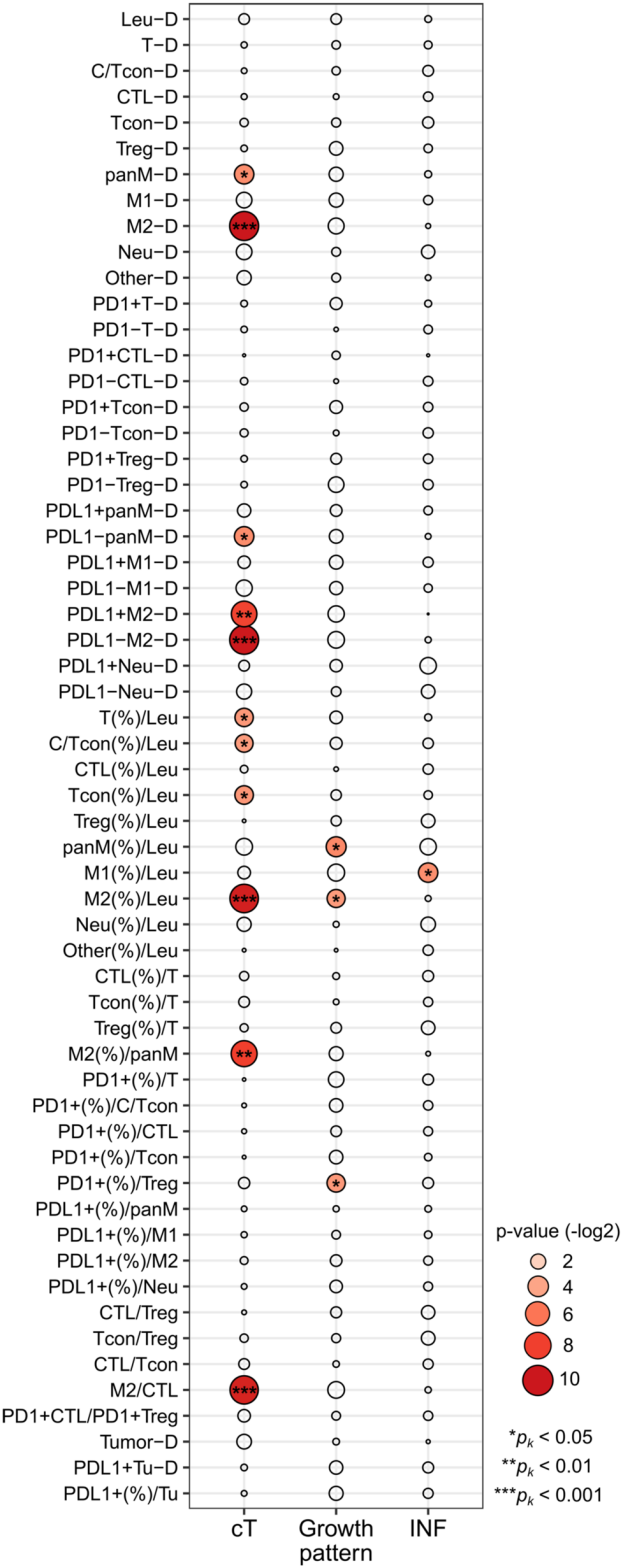
Comparative analyses of the clinicopathological characteristics by immune parameter. Comparative analyses of three cT grades (cT1, cT2, and cT3/T4), three clinical growth patterns (superficial, exophytic, and endophytic), and three INF classifications (INFa, INFb, and INFc) of the 58 immune parameters were conducted using the Kruskal–Wallis test. Differences in *p*_*k*_ values are shown by changes in color and size

**Fig. 3:**
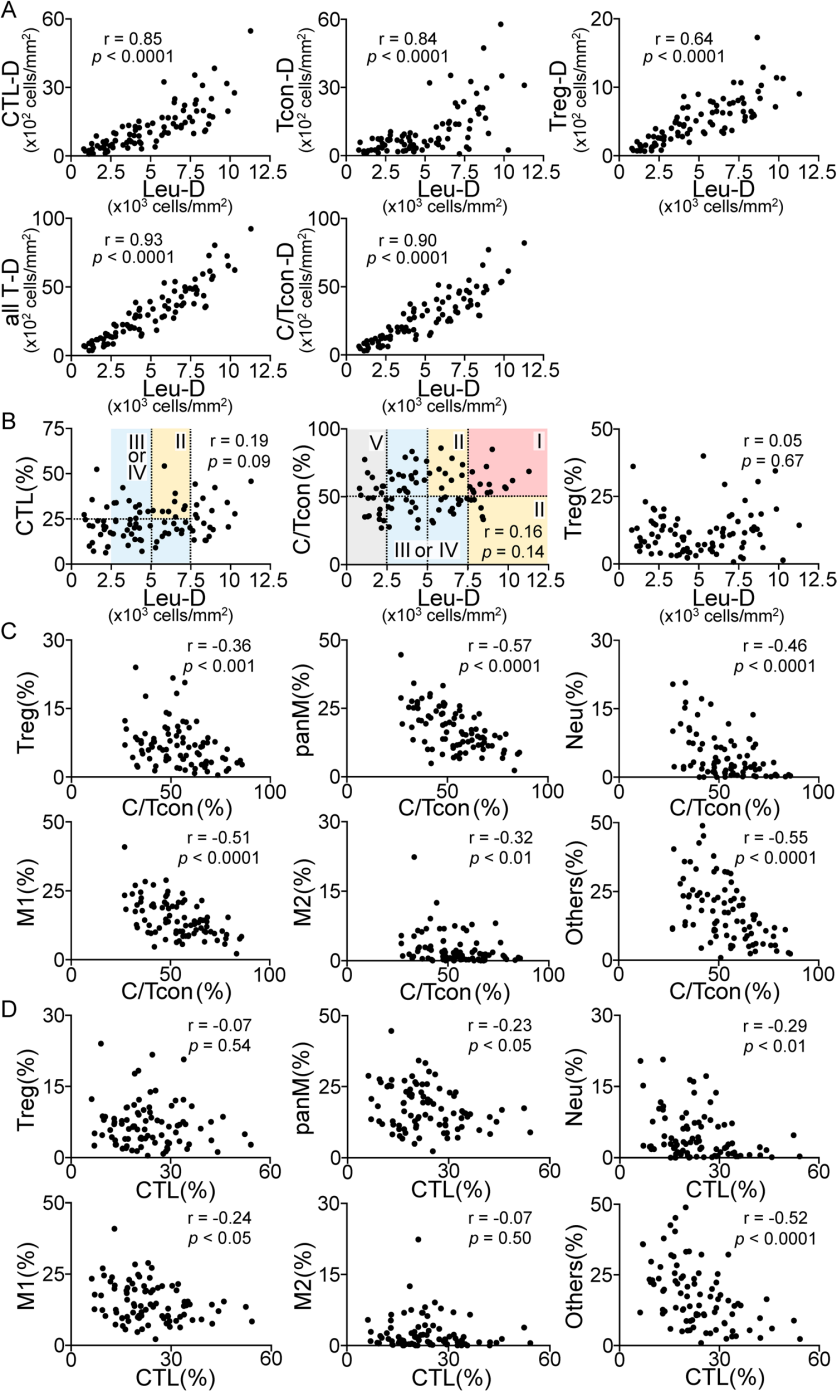
Correlations between immune parameters. Positive **A**, absent **B**, and negative (**C** and **D**) correlations. r; correlation coefficient. D, density (cells/mm^2^). Leu, CD45^+^ leukocytes; CTL, CD8^+^ cytotoxic T lymphocytes; Tcon, CD4^+^Foxp3^–^ conventional T cells; Treg, CD4^+^Foxp3^+^ regulatory T cells; C/Tcon, CTL plus Tcon; all T, C/Tcon plus Treg; panM, CD68^+^ pan-macrophages; M1, CD68^+^CD163^–^ M1-like macrophages; M2, CD68^+^CD163^+^ M2-like macrophages; Neu, CD66b^+^ neutrophils; Others, non-T and non-myeloid CD45^+^ cells

The characteristics of the 87 therapy-naïve TSCC patients are shown in Table 1. We analyzed the immune profiles of the tumor stroma regions at the invasive fronts. We assessed the correlations of a total of 58 distinct immune parameters [12] (Fig. 1) and compared the immune parameter values against the T stages, growth patterns, or INF classifications (Fig. 2). The density of CD45^+^ leukocytes (Leu-D) correlated positively with the densities of three T-cell subsets (CD8^+^ CTL, Tcon, and Treg), all T-cells, and CTL plus Tcon (C/Tcon) (Fig. 3A). No correlation was apparent between Leu-D status and the proportions of the aforementioned immune subsets (Figs. 1 and 3B). Therefore, we considered that Leu-D status serves as a primary index for evaluating active innate and adaptive immune responses mediated by all myeloid cells and lymphocytes. The mixed fraction of CTL and Tcon (C/Tcon) was assumed to comprise anti-tumor effector cells. The C/Tcon proportion (%) was negatively correlated with the percentages of Treg, panM, M1, M2, Neu, and Others (Fig. 3C). Consistent with our previous report [12], samples of early T stages exhibited clearly reduced M2-related indices [M2-D, M2(%), M2(%)/panM] in addition to a lower panM-D index (Supplementary Fig. 1A). The group exhibiting an endophytic growth pattern, associated with poorer prognosis, had the highest panM(%) and M2(%) values (Supplementary Fig. 1B). The INFc group, which exhibited more severe invasion, had the highest M1(%) values (Supplementary Fig. 1C). These results suggest that the abovementioned immune subsets (Treg, panM, M1, M2, Neu, and Others) may negatively impact anti-TSCC immune responses.

**Table 1:** Characteristics of 87 therapy-naïve TSCC patients

Characteristics	T stage			
	T1	T2	T3	T4
	43 (49.4%)	33 (37.9%)	10 (11.5%)	1 (1.2%)
N stage				
N0	42	28	7	0
N1-2	1	5	3	1
Growth pattern				
Superficial	23	11	3	0
Exophytic	8	6	2	0
Endophytic	12	16	5	1
INF (YK) classification				
INFa (YK-1/2)	17	11	2	0
INFb (YK-3)	16	12	5	0
INFc (YK-4C/4D)	10	10	3	1
R/M				
Local recurrence	1	0	1	0
Node metastasis	6	1	2	0
5-year DFS (%)	83.7 (36/43)	97.0 (32/33)	70.0 (7/10)	100 (1/1)

DFS: Disease free survival

### Stratification of TSCC immunotypes

**Fig. 4:**
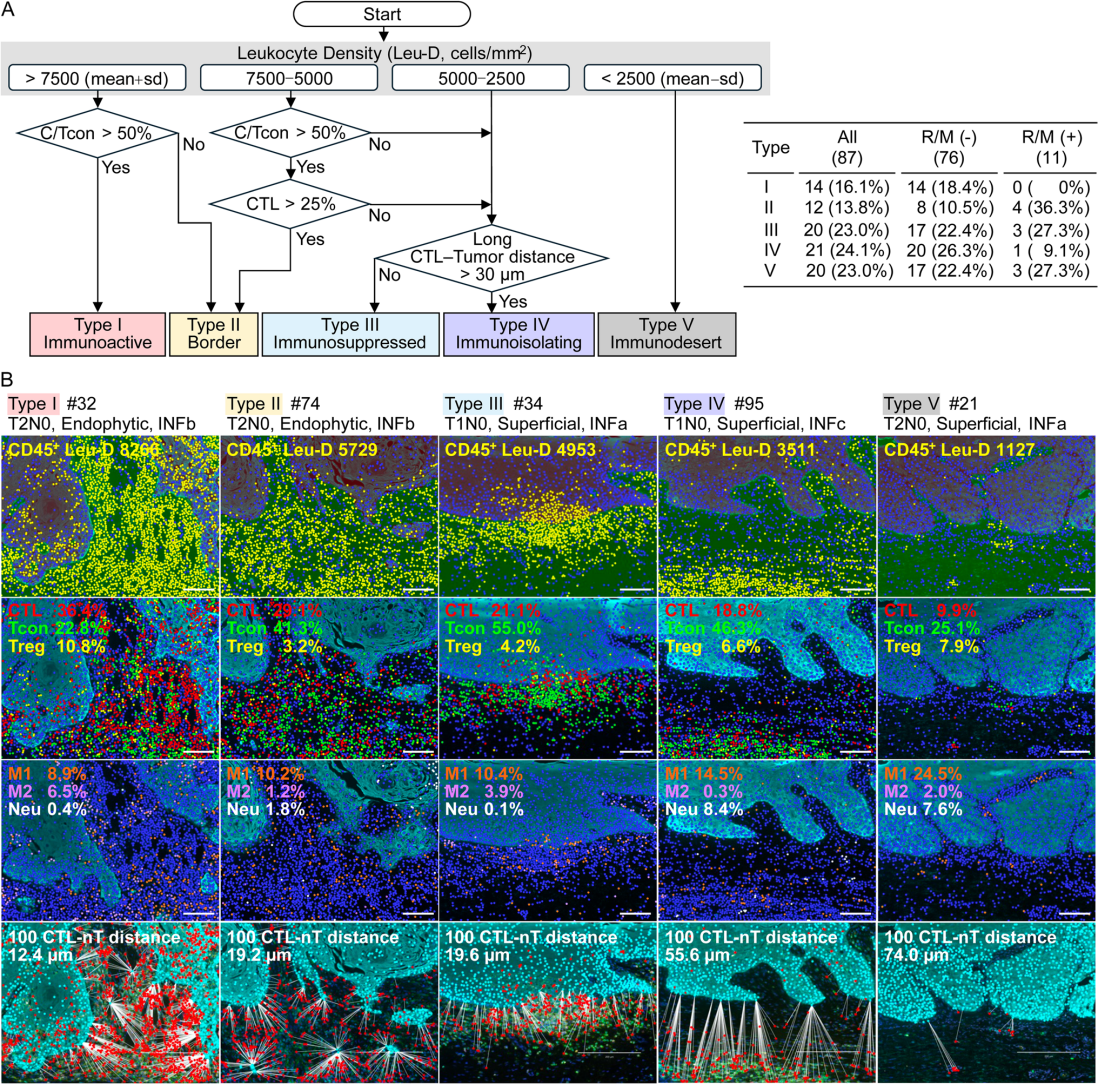
Stratification of TSCC immunotypes. **A** Flow chart of immunotype stratification and the characteristics of the immunotypes. **B** Representative images of each immunotype. Upper panels, CD45^+^ Leu (yellow); second-row panels, three T cell subsets–CTL (red), Tcon (green), and Treg (yellow); third-row panels, myeloid cell subsets–M1 (orange), M2 (purple), and Neu (white). All percentages are those of CD45^+^ leukocytes. Scale bars: 100 µm. Bottom panels: The distance from each CTL to the nearest tumor cell (nT). The values are the means of 100 CTL-nT distances. CK^+^ tumor cells (cyan) and CTL (red) are shown. White lines show the CTL-to-nT connections. Scale bars: 200 µm

**Fig. 5:**
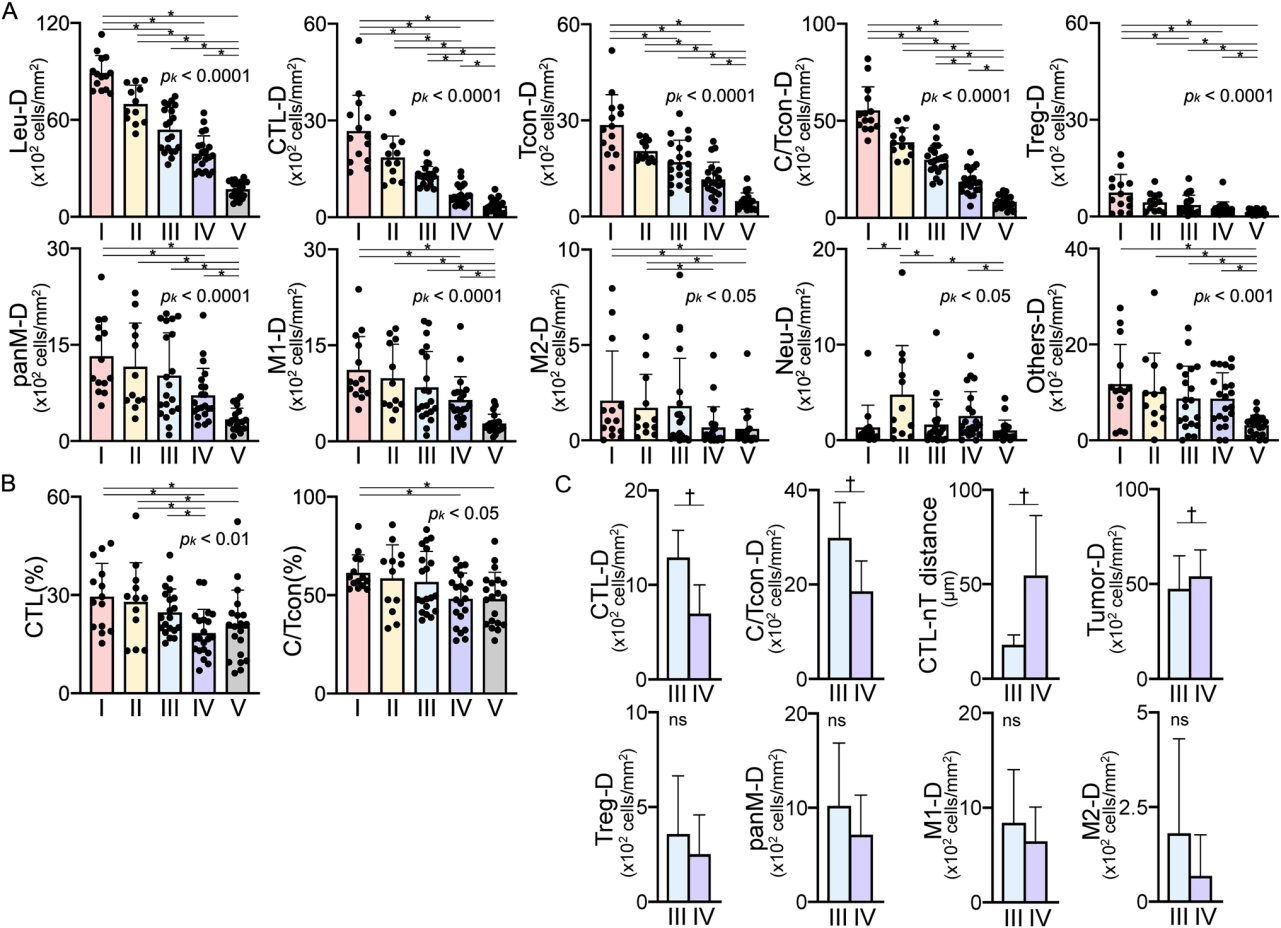
Immune parameters by immunotype. Densities **A** and percentages **B** are shown. The Kruskal–Wallis test was performed, followed by the uncorrected Dunn test (*p < 0.05). **C** Comparison between type III and IV. The Mann–Whitney U test was performed. ^*†*^*p* < 0.05. ns, Not significant. Bars: mean ± SD

When classifying the immunotypes, we initially divided all 87 cases into four groups based on the Leu-D magnitude, as shown in a flow chart (Fig. 4A). In the top two groups, i.e., those where the Leu-D level exceeded the mean + 1 standard deviation (SD) (> 7500 cells/mm^2^) and the level was between the mean and the mean + 1 SD (5000–7500 cells/mm^2^), an above-average C/Tcon(%) or CTL(%) served as the second or third criterion when assessing active anti-tumor T-cell immunity (types I and II). Cases where the Leu-D level was lower than the mean–1 SD (< 2500 cells/mm^2^) were classified as immunodesert type (type V). When we examined immune profiles of additional 41 cases excluding types I, II, and V, we realized apparent differences in the spatial immune cell distribution. Spatial relationships between CD8^+^ T cells and head and neck tumor cells was associated with ICI response [18]. Thus, the distances between 100 CTL and the nearest tumor cells (100 CTL-nT) were assessed by spatial image analysis (bottom panels in Figs. 4B and 5C). The cut-off 100 CTL-nT distance between type III and IV was assessed using both the mean and median values. Cases with a 100 CTL-nT distance > 30 µm and visually verified for immuno-isolating band-like stroma-rich zones were defined as immunoisolating type (type IV) (Fig. 4A). The remaining cases, which exhibited moderate Leu-D levels but lower CTL proportions, were defined as type III. Of 87 TSCC, the immunoactive type I, border type II, immunosuppressed type III, immunoisolating type IV, and immunodesert type V numbers (proportions) were 14 (16.1%), 12 (13.8%), 20 (23.0%), 21 (24.1%), and 20 (23.0%) cases, respectively (Fig. 4A). Of the 11 R/M cases among the 87 TSCC patients, none were type I. Representative images of each type (Fig. 4B) and pie charts of the immune profiles by immunotype (Supplementary Fig. 2A) are shown.

### Immunological characteristics of five TSCC immunotypes

The densities of all leukocytes, and of the T-cell and macrophage subsets (Leu, CTL, Tcon, C/Tcon, Treg, panM, M1, and M2) gradually decreased in numerical order of immunotype (Fig. 5A), but the percentages did not (Supplementary Fig. 3). By density, type I was the highest in terms of (positive) anti-tumor immune cell infiltration, and type V was the lowest of all immune subsets. Both types I and II exhibited higher Leu-, CTL-, and C/Tcon-D values than the other three types. Type II exhibited lower CTL- and Tcon-D values but a higher Neu-D value than type I. In contrast to types I and II, types III–V had certain immune regulatory properties. Comparison between type III and IV revealed that CTL- and C/Tcon-D were significantly higher in type III, and the CTL-nT distance and tumor-D were significantly greater in type IV (Fig. 5C). The densities of immunoregulatory Treg, panM, M1, and M2 were higher in type III, albeit not significantly different.

### PD-1 and PD-L1 expression are closely linked during infiltration of macrophages and CTL

**Fig. 6:**
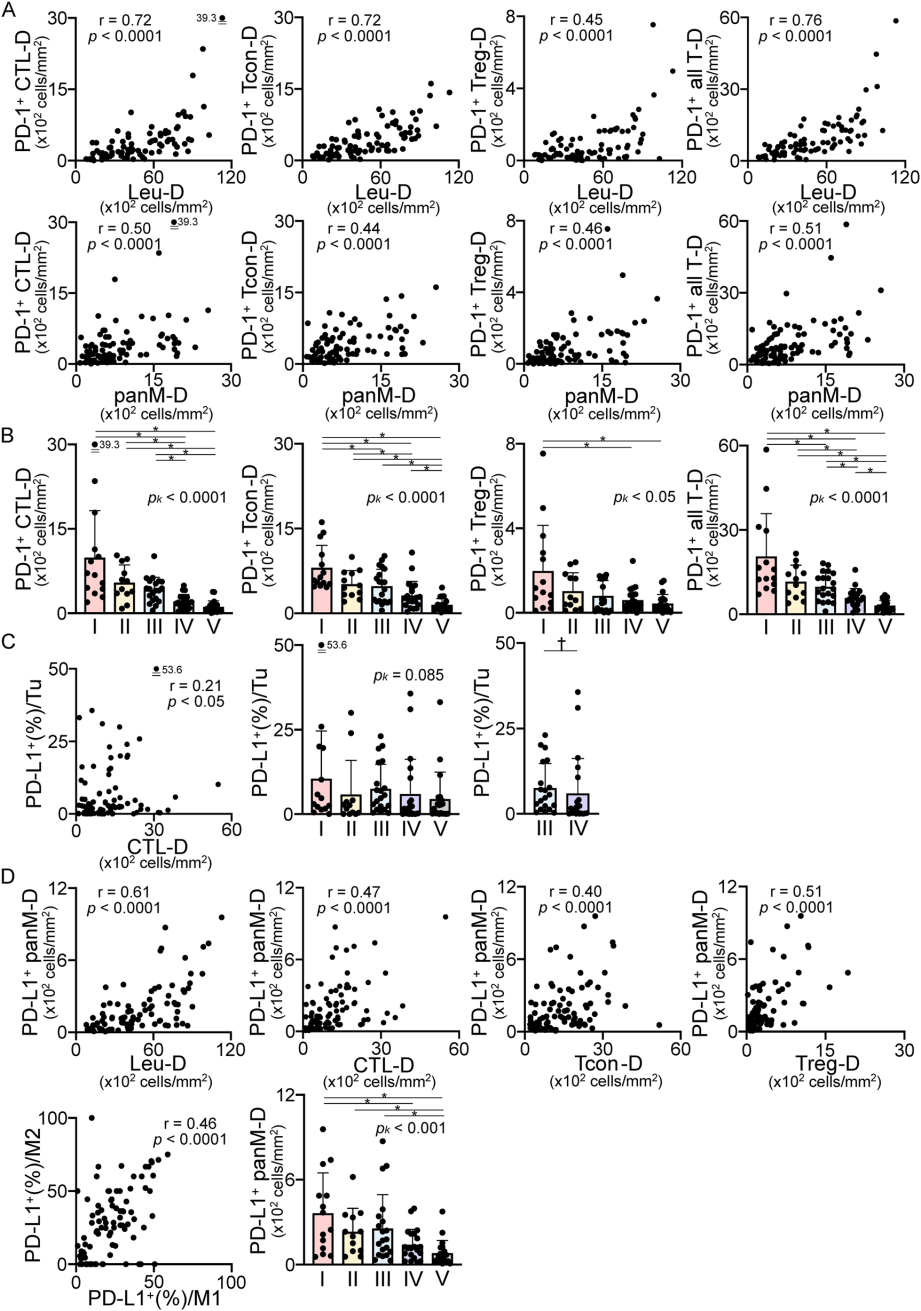
Correlations and comparative analyses of PD-1 and PD-L1 expression. Correlations of PD-1^+^ T-cell subsets **A**. PD-1^+^ T-cell subsets by immunotype **B**. Correlations and comparisons of PD-L1^+^ tumor cells **C** and PD-L1^+^ macrophages **D**. r, correlation coefficient. When comparing five and two immunotypes, the Kruskal–Wallis test followed by the uncorrected Dunn test (*p < 0.05) and the Mann–Whitney U test (^*†*^*p* < 0.05) were performed. Bars: mean ± SD

The densities of PD-1-expressing T cells (CTL, Tcon, Treg, and all T cells) were positively correlated with Leu- and panM-D status (Fig. 6A). The PD-1^+^(%)/CTL or PD1^+^(%)/Treg values were not correlated with the CTL density (CTL-D), Tcon-D, and C/Tcon-D (Supplementary Fig. 4A). By immunotype, type I had the highest PD-1 density in any T-cell subset, and type V was the lowest (Fig. 6B).

PD-L1(%) of CK^+^ tumor cells (Tu) was highly variable (6.7 ± 10.0%, 0.0–53.6%). The expression level and distribution varied by region even in the same individuals, consistent with previous observations [12, 19]. PD-L1^+^(%)/Tu was positively correlated with CTL-D (Fig. 6C), but not with Leu-D, Tcon-D, or Treg-D (Fig. 1). PD-L1^+^(%)/Tu was not clearly correlated with the immunotype, but PD-L1^+^(%)/Tu in type III was higher than that in type IV (Fig. 6C). Unlike PD-L1^+^(%)/Tu, PD-L1^+^(%)/panM was relatively high (24.0 ± 14.7%, 2.4–59.2%). PD-L1^+^(%) was closely correlated between M1 and M2 (Fig. 6D) despite the marked difference between the M1 and M2 densities (Fig. 5A). PD-L1^+^ panM-D was positively correlated with the Leu-, CTL-, Tcon-, and Treg-D values (Fig. 6D). PD-L1^+^ panM-D was correlated with the immunotype grade. The type I–III values were markedly higher than those of type V (Fig. 6D). Similar to PD-1 expression, type I cases expressed high PD-L1 levels, whereas type IV and V cases had lower levels. These data suggest that PD-1^+^ T-cell distribution is dependent on macrophage recruitment, and that a wide PD-L1^+^ macrophage distribution requires CTL recruitment.

### Immune profiles and immunotypes of PD-1 ICI-treated TSCC patients

We retrospectively examined the immune profiles of 17 TSCC patients who received PD-1 ICI treatments. The clinicopathological data reflecting the best ICI response are summarized in Table 2. Of the 17 cases, 1 (5.9%) and 2 (11.7%) experienced a complete response (CR) or partial response (PR), with 1 case of stable disease (SD). The overall response rate was 17.6%. Individual data, including the immunotypes, are shown in a heatmap (Supplementary Fig. 5) and pie charts display the immune profile (Supplementary Fig. 2B). Despite not responding ICIs, three patients (#24 PD, type III; #63 CR, type V; #67 PR, type II) were alive after 3 years without disease. One CR case (#63) had received 5-fluorouracil-plus-cisplatin chemotherapy before nivolumab monotherapy, and the other PR (#67) and PD (#24) had been prescribed cetuximab/paclitaxel therapy after nivolumab monotherapy, resulting in CR. The type I–V immunotype classifications had 0 (0%), 2 (11.8%), 5 (29.4%), 2 (11.8%), and 8 (47.0%) cases, respectively. There was no type I case. Most cases (88.2%) were immune-regulated types III–V, and almost half (47%) were type V.

**Table 2:** Characteristics of 17 ICI-treated TSCC patience

Characteristics	ICI response			
	CR	PR	SD	PD
	1 (5.9%)	2 (11.7%)	1 (5.9%)	13 (76.5%)
*Clinical outcome*				
PFS (month)	65.7*	12.6*, 16.0	2.7	2.2 ± 1.5 (0.2–5.3)
3-year OS (%)	1 (100)	2 (100)	0 (0)	2 (15.4)
3-year DFS (%)	1 (100)	1 (50)	0 (0)	1 (7.7)
*Clinicopathological data*				
TN stage				
T1		N0 (1)	N0 (1)	N0 (2)
T2				N0 (2), N1 (1)
T3		N2 (1)		N0 (1), N1–2 (2)
T4	N1 (1)			N0 (2), N1–3 (3)
Growth patterm				
Superficial	0	0	1	3
Exophytic	0	1	0	1
Endophytic	1	1	0	9
INF (YK) classification				
INFa (YK-1/2)	0	0	0	0
INFb (YK-3)	0	1	1	4
INFc (YK-4C/4D)	1	1	0	9

PFS: Progression-free survival; OS: Overal survival; DFS: Disease-free survival

*Withdrawn at this time point

### PD-1 and PD-L1 expression in ICI-treated TSCC patients

**Fig. 7:**
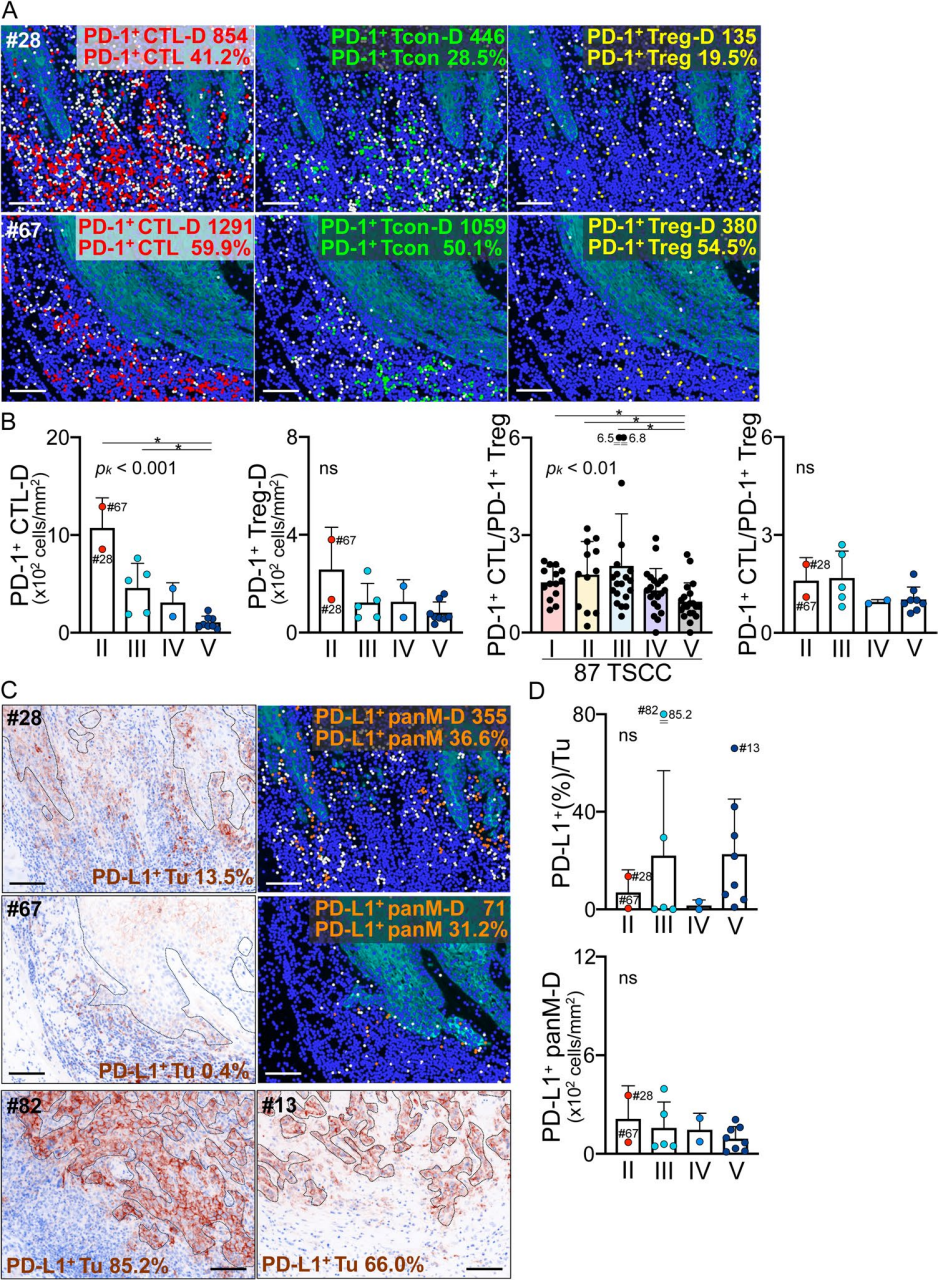
PD-1 and PD-L1 expression levels in ICI-treated TSCC patients. **A** Images showing PD-1 expression in two type II cases. PD-1^+^ CTL (red), PD-1^+^ Tcon (green), PD-1^+^ Treg (yellow), and PD-1^–^ cells in each T-cell subset (white). Scale bars: 100 µm. Comparative analyses of PD-1^+^ T cells **B** and PD-L1^+^ tumors and macrophages **D**. The Kruskal–Wallis test was performed, followed by the uncorrected Dunn test. *p_*k*_ < 0.05. ns; not significant. **C** Representative images showing PD-L1 expression in two type II cases and two high tumor PD-L1 cases. PD-L1 expression (brown) is revealed by the component image. Black lines show the segmented borders between tumors and stromal areas. Dots in the right upper and middle panels: PD-L1^+^ panM (orange) and PD-L1^–^ panM (white). Scale bars: 100 µm

Two type II ICI cases (#28 and #67) exhibited higher densities and percentages of PD-1^+^ CTL and Tcon, but case #67 had a high Treg PD-1 level (Fig. 7A and B). The PD-1^+^ CTL-D values of types II and III were markedly higher than those of type V. A higher PD-1^+^ CTL/PD-1^+^ Treg ratio revealed PD-1 by genomic analyses served as a promising biomarker of a PD-1 ICI response in patients with several solid cancers [20]. In 87 TSCC patients, the PD-1^+^ CTL/PD-1^+^ Treg ratios in type I–III were significantly higher than in type V, but we found no clear difference by immunotype in the 17 patients in the ICI cohort (Fig. 7B). Images showing the PD-L1 expression of type II cases (#28 and #67) and another two cases (#82 and #13) with high tumor-PD-L1 levels are shown in Fig. 7C. As mentioned above (Fig. 6C), the tumor-PD-L1 proportions were highly variable, and the percentages were low except in two cases (#82 and #13) with very high proportions and expression levels (Fig. 7D). In such cases, there may be intrinsic PD-L1 induction mechanisms, related to tumor carcinogenesis [21]. PD-L1^+^ panM-D did not clearly correlate with any immunotype (Fig. 7D). The #67 type II case exhibited the highest PD-1^+^ CTL-D, but low PD-L1, in both tumor cells and macrophages. As stated above, most cases exhibited imbalanced PD-1 and PD-L1 expression. Cases expressing high levels of both PD-1 and PD-L1, for whom ICIs are appropriate, were rare.

## Discussion

We identified five TSCC immunotypes (immunoactive, border, immunosuppressed, immunoisolating, and immunodesert-types) via comprehensive immune profiling analyses at the invasive tumor fronts of 87 therapy-naïve specimens obtained during surgical resection.

M1 have been generally viewed as anti-tumor macrophages. However, here, the M1 subset exerted a negative impact, as did the M2, Treg, and Others. We and others have demonstrated that CD163^+^ M2-like tumor-associated macrophages (TAMs) affect the prognosis of HNSCC [12, 22], but the roles played by CD163^–^ TAMs in antitumor responses remain unclear. Spatial TAM heterogeneity among the various tumor sites, namely the “invasive front”, “tumor nest”, and “stroma” may be associated with differences in the effects of TAMs that interact with tumor, stroma, and immune cells [23]. As both M1 and M2 expressed relatively high levels of PD-L1, the interactions of PD-L1^+^ M1 with PD-1^+^ C/Tcon would downregulate the effector functions of T cells, having a negative impact on anti-TSCC responses.

When classifying the immunotypes, we included the spatial distance between CTL and tumor cells when separating type III from IV. A previous four immunotype classification based on CD8^+^ T cell density alone in HNSCC did not include spatial assessment [7]. In addition to the density and frequency evaluation, understanding of spatial organization is important for tumor progression and treatment responses [18, 24, 25]. ICI non-responders demonstrated a significantly higher distance between CTL and tumor cells [18, 24]. In type IV cases, we observed band-like stroma rich zone without T-cell infiltration. Alternations of the stromal architecture by tumor cells, tumor-associated endothelial cells, and cancer-associated fibroblasts (CAFs) prevent the recruitment of CTL to tumor cells [26]. CAFs restrict CTL actions by secreting transforming growth factor beta (TGF-β) or IL-6 [25, 27]. In contrast, the presence of immunoregulatory Treg and TAMs near tumor cells may directly attenuate the recruitment and effector functions of CTL in type III patients.

Turning to PD-1 and PD-L1 evaluation, positive correlations were observed between the panM-D and PD-1^+^ CTL-D values, and between the CTL-D and PD-L1^+^ panM-D values (Fig. 6). PD-1 expression on exhausted T cells (Tex) is regulated by multiple transcription factors that related to T-cell activation signaling [28]. Antigen-specific CD8^+^ T cells become preferentially localized in TAM-rich areas and then engage in long-lasting, strong interactions that impede CD8^+^ T cell migration to tumor nets [29]. Chemokines secreted by PD-1^+^ Tex attract monocytes to the TME, and TAMs then accumulate. Reciprocally, TAMs contribute to the exhaustion programs of CD8^+^ T cells [30]. Thus, both TAMs and Tex are spatiotemporally co-dependent in the TME.

Immunoactive type I and border type II are equivalent to the “hot”, “CD8-dominant”, or “fully infiltrated” [7] subtype characterized by abundant Leu and CTL infiltration. We found that high CTL-D correlated with elevated PD-1 and PD-L1 levels. A high CTL density correlated with strong PD-L1 expression was associated with better survival of HPV-negative HNSCC patients [31]. A high number of PD-1^+^CD8^+^ T cells was an independent prognostic factor in patients of oral cavity and oropharyngeal SCCs [32]. When CD8 and PD-L1 were stained separately, stromal high CD8^+^ T cells together with high PD-L1 had a better prognosis in nivolumab-treated HNSCC patients [33]. Together, the data suggest that a high CTL-D may be the optimal biomarker for better prognosis and ICI response, because high PD-1 and PD-L1 levels were linked to high Leu-D and high CTL levels. Although we had no type I in our 17 ICI-treated cases, type I TSCC may be sensitive to PD-(L)1 ICI monotherapy. Type II TSCC may require additional ICIs that target CTLA-4, TIM3, or LAG3 to convert Tex into effector T cells, thereby enhancing CTL function [34]. Immunotypes III–V are “cold” tumors characterized by low Leu- and CTL-D values. Most TSCCs (~ 70%) are of these types, which may be resistant to ICI treatment and thus require combined therapies based on the immunotype. We summarized immunological characteristics, prediction of ICI response, and possible combined treatments in each immunotype in Supplementary table. In type III patients, immunoregulatory cells such as Treg, TAMs, Neu, and Others must be targeted. CCR4 antagonists (such as mogamulizumab) that preventing Treg recruitment [35], and/or CSF-1R inhibitors or multispecific CSF1R/CCR2/TGF-β antibody that inhibit TAMs generation and recruitment [36, 37], may be combined with a PD-(L)1 ICI. In type IV patients, stromal CAFs, angiogenesis, and tumor cells must be carefully monitored. EGFR inhibitors (e.g., cetuximab, gefitinib), VEGFR inhibitors (e.g., bevacizumab) [38], TGF-βR inhibitors [39], a dual TGF-βRII/PD-L1 inhibitor (bintrafusp alfa) [40], and/or signal transducer and activator of transcription 3 (STAT3) inhibitors [41] are treatment candidates. The Leu level of the immunodesert type V is very low, possibly because of a lack of immunoreactive tumor antigens (neoantigens) [42]. As most Japanese cases are not associated with HPV infection [43], and exhibit markedly low tumor mutation burdens [44, 45], immunotherapy may be ineffective without tumor antigen modification.

Our study has some limitations. We could not verify the clinical prognosis (survival and R/M frequency) by immunotype because we enrolled only therapy-naïve TSCC cases. Thus, the clinical outcome of 87 TSCC cohort was very good and the case number of ICI-treated TSCC study was too small to validate. Further studies are required. We used two panels of six-color mIF and tissue imaging systems, but ultimately found that the crucial information is the infiltration and distribution of CD45^+^, CD8^+^, and tumor cells. Thus, trained pathologists can classify five immunotypes by evaluating the hematoxylin and eosin (H&E) and single- or double-color anti-CD45 and CD8 antibody staining profiles. We hope that our TSCC-specific immunotype classification will become routine and be included in TSCC treatment guideline. Our classification will aid personalized treatments, especially immunotherapies, which improve clinical outcomes.

## Supplementary Information

Below is the link to the electronic supplementary material.

Supplementary file1 (PDF 2757 KB)

## Data Availability

The data supporting the results reported in this study can be made available upon reasonable request to the corresponding author.

## Abbreviations

CTL: Cytotoxic T lymphocytes
C/Tcon: CTL plus Tcon
CAF: Cancer-associated fibroblast
D: Density
Leu: Leukocytes
M1: M1-like macrophages
M2: M2-like macrophages
Neu: Neutrophils
panM: Pan-macrophages
R/M: Recurrent and metastatic
TSCC: Tongue squamous cell carcinoma
TME: Tumor microenvironment
Tcon: Conventional T cells
Treg: Regulatory T cells
Tu: Tumor cells
TAM: Tumor-associated macrophage
Tex: Exhausted T cells
