# Supplementary material for: Stratification of the immunotypes of tongue squamous cell carcinoma to improve prognosis and the response to immune checkpoint inhibitors
Source: Cancer Immunol Immunother. 2025 Mar 1;74(4):130. doi: 10.1007/s00262-025-03982-9 (PMC11872838; doi:10.1007/s00262-025-03982-9)
Supplement: Supplementary file 1 — Supplementary file1 (PDF 2757 KB) [file 262_2025_3982_MOESM1_ESM.pdf]

## Supplementary Materials

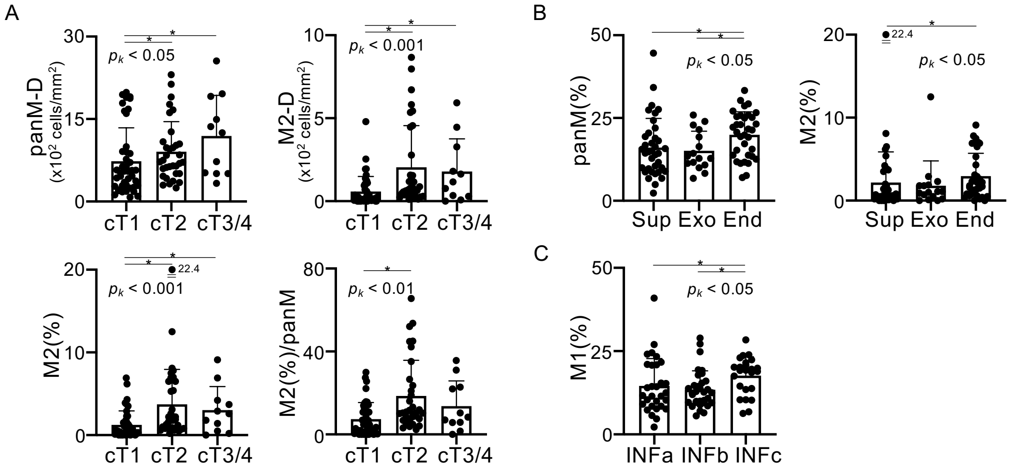


**Supplementary Fig. 1** Comparison of macrophage-related indices .

The indicated macrophage-related immune parameters were compared among groups according to clinical T stage (cT) (A), clinical growth pattern (B), and INF classification (C). The Kruskal–Wallis test was performed, followed by the uncorrected Dunn test (*$p < 0.05$). Clinical growth patterns: Sup (superficial), Exo (exophytic), and End (endophytic).

## Supplementary Fig. 2

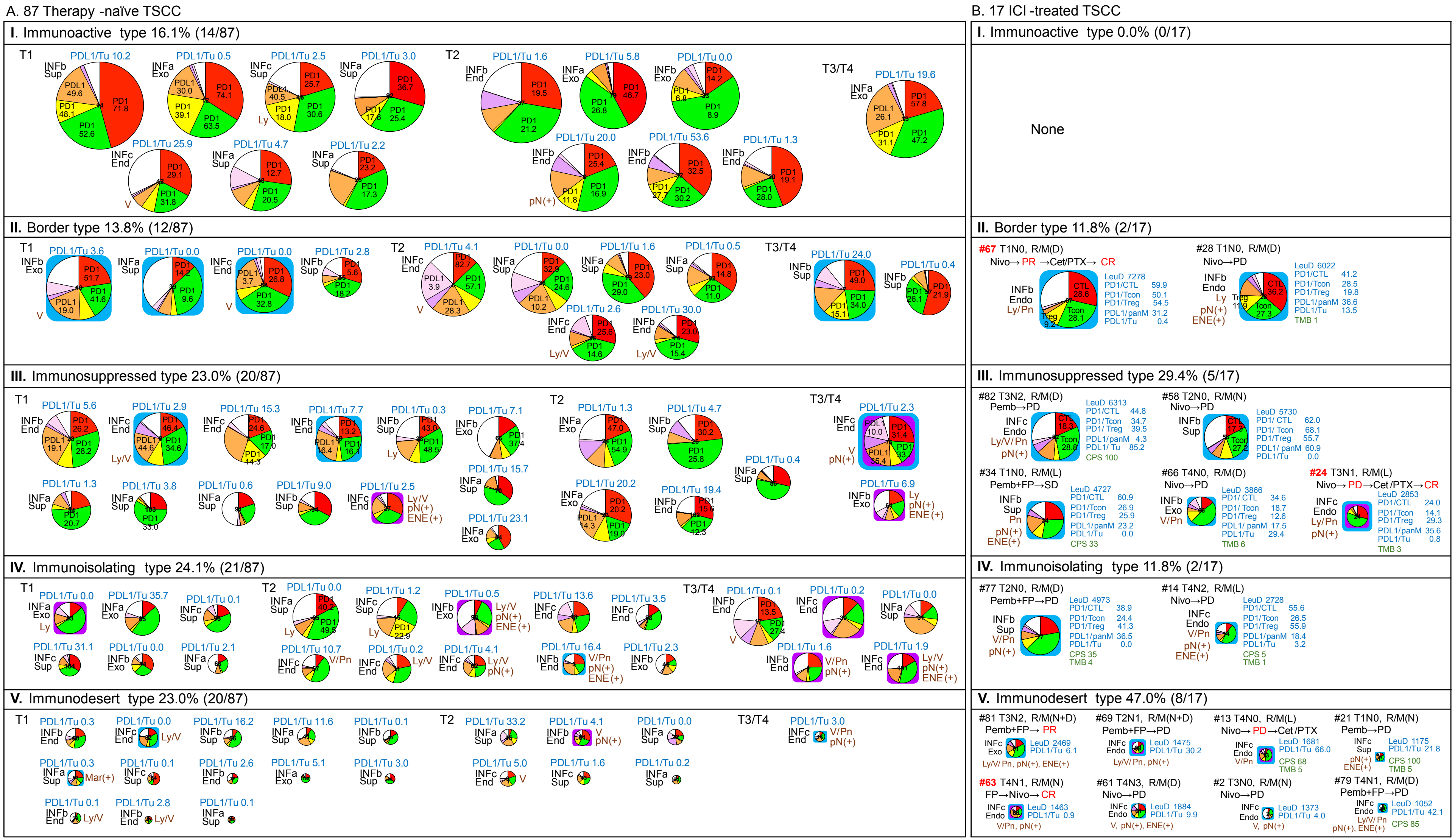

**Supplementary Fig. 2** TSCC immune profiles by immunotype.

Immune profiles of 87 therapy-naïve (A) and 17 ICI-treated (B) TSCC patients. The placement of each pie chart is based on the immunotype classification. The "pie chart diameters" indicate $CD45^+$ Leu density values and relative proportions of CTL (red), Tcon (green), Treg (yellow), M1 (orange), M2 (purple), Neu (pink), and Others (white) are shown. Cases that developed R/M status and received adjuvant therapy are indicated by blue and purple squares, respectively. In (A), the values within the pie chart are the percentages of $PD\text{-}1^+$ or $PD\text{-}L1^+$ cells. The proportions (%) of $PD\text{-}L1^+$ cells within $CK^+$ tumor cells (PDL1/Tu) are shown in blue. Clinical growth patterns: Sup (superficial), Exo (exophytic), and End (endophytic); pathological invasion: Ly (lymphatic vessel), V (venous), Pn (perineural), ENE (extranodal extension), pN (pathological node metastasis). Mar (+): margin-positive. In (B), Leu-D, $PD\text{-}1^+$ cell (%) within the indicated T-cell subsets, $PD\text{-}L1^+$ (%) within panM (PDL1/panM) or Tu cells (PDL1/Tu), TMB (tumor mutation burden) and CPS (combined positive score) are shown on the right. R/M (L), local recurrence; R/M (N), lymph node metastasis; R/M (D), distant metastasis. The flow of clinical treatments and outcomes are shown in the upper regions of the pie charts. Nivo, Nivolumab; Pemb, Pembrolizumab; Cet, Cetuximab; PTX, Paclitaxel; FP, 5-Fluorouracil plus Cisplatin.

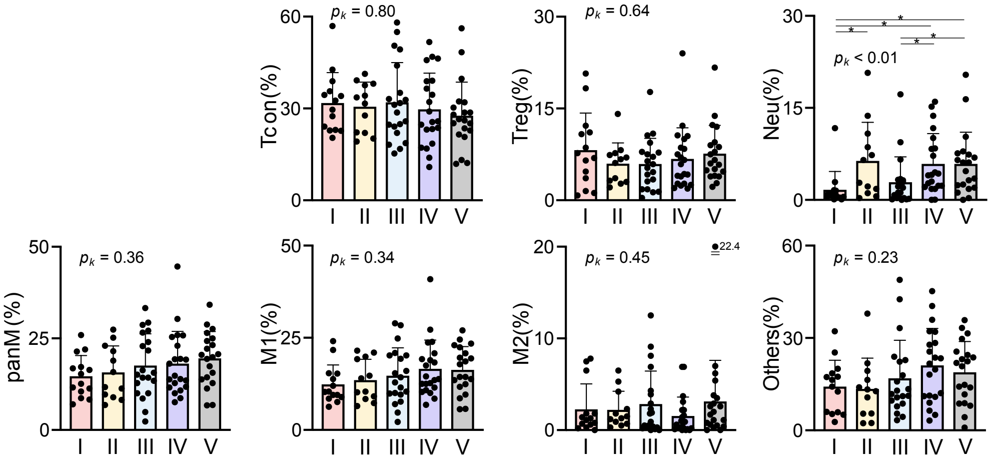


**Supplementary Fig. 3** Immune parameters by immunotype. The Kruskal–Wallis test was performed. Bars: mean ± SD.

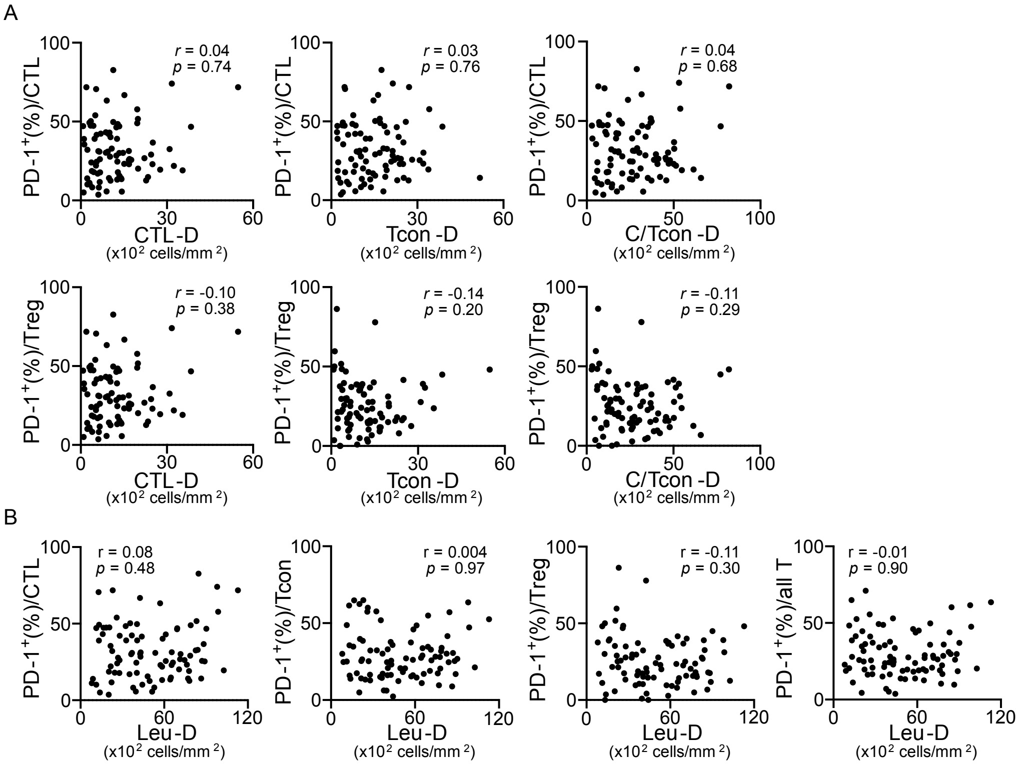


**Supplementary Fig. 4** Correlations between the percentages of PD-1$^+$ T cell subsets and the densities of T cell subsets. No correlations were observed among the percentages of PD-1$^+$ T-cells and the CTL-, Tcon-, and C/Tcon-D values (A), or between the percentages of PD-1$^+$ T-cells and the Leu-D values (B). Spearman's rank correlations were conducted. r, correlation coefficient.

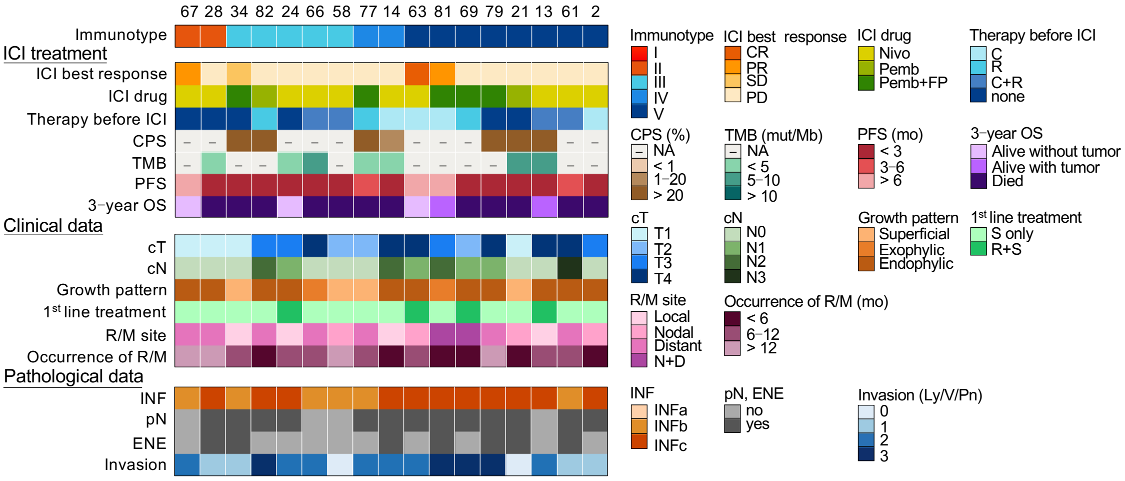


**Supplementary Fig. 5** Heatmap of data of the 17 ICI-treated TSCC patients.

All ICI treatment-related issues and clinicopathological characteristics are shown. S, surgery; C, chemotherapy; R, radiotherapy; CPS, combined positive score; TMB, tumor mutation burden; PFS, progression-free survival; OS, overall survival; pN, pathological node metastasis; ENE, extranodal extension; Ly, lymphatic vessel; V, venous; Pn, perineural. NA, not available.

**Supplementary Table**

Immunological characteristics, PD-1 ICI sensitivity, and possible combined treatments by immunotype

| Immunotype | Immunological characteristics | PD-1 ICI sensitivity | Possible combined treatments |
|---|---|---|---|
| Type I | High CTL-D<br>High PD-1/PD-L1 | Sensitive | Not required |
| Type II | CTL-D (Type I > II)<br>PD-1/PD-L1 (Type 1 > II) | Less sensitive | Targeting additional ICIs<br>+ anti-CTLA-4<br>+ anti-TIM-3 or anti-LAG3 |
| Type III | Moderate~low CTL<br>CTL-D (Type III > IV)<br>**Immunoregulatory cell-dominant status**<br>(Treg, TAMs, Neu, Others) | Resistant | Targeting Treg recruitment<br>+ CCR4 antagonists (Mogamulizumab)<br>Targeting TAMs generation and recruitment<br>+ CSF1R inhibitors (Emactuzumab, Pexidartinib)<br>+ multispecific CSF1R/CCR2/TGF-β Ab<br>Targeting TAN/PMN-MDSC<br>+ Gemcitabine<br>+ IDO1 inhibitors |
| Type IV | Moderate~low CTL<br>Long CTL–tumor distance<br>**Alterations of stromal architecture** | Resistant | Targeting CAFs /Tumor cells /Endothelial cells<br>+ EGFR inhibitors (Cetuximab, Gefitinib)<br>+ VEGFR inhibitors (Bevacizumab )<br>+ TGF-βR inhibitors (Vactosertib)<br>+ dual TGF-βRII/PD-L1 inhibitor (Bintrafusp alfa)<br>+ STAT3 inhibitors |
| Type V | No antitumor response<br>**Lack of tumor antigens (neoantigens)** | Resistant | Require tumor-Ag modification |

TAMs: tumor-associated macrophages, TAN: tumor-associated neutrophils, MDSC: myeloid-derived suppressor cells, CSF1R: colony-stimulating factor 1 receptor, PMN: polymorphonuclear, IDO: indoleamine 2,3-dioxygenase, EGFR: epidermal growth factor receptor, VEGFR: vascular endothelial growth factor receptor, TGF-β: transforming growth factor beta, STAT3: signal transducer and activator of transcription 3.
